# RORγt-expressing cells attenuate cardiac remodeling after myocardial infarction

**DOI:** 10.1371/journal.pone.0183584

**Published:** 2017-08-21

**Authors:** Daichi Enomoto, Kotaro Matsumoto, Tomomi Yamashita, Arisa Kobayashi, Makiko Maeda, Hiroyuki Nakayama, Masanori Obana, Yasushi Fujio

**Affiliations:** Laboratory of Clinical Science and Biomedicine, Graduate School of Pharmaceutical Sciences, Osaka University, Suita, Osaka, Japan; Albert Einstein College of Medicine, UNITED STATES

## Abstract

**Aims:**

Retinoic acid receptor-related orphan nuclear receptor γt (RORγt) is a transcriptional factor responsible for IL-17-producing T-cell differentiation. Although it was demonstrated that RORγt plays essential roles in the onset of autoimmune myocarditis, pathophysiological significance of RORγt in cardiac remodeling after myocardial infarction (MI) remains to be fully elucidated.

**Methods and results:**

MI was generated by ligating coronary artery. The expression of RORγt and IL-17A transcripts increased in murine hearts after MI. Additionally, immunohistochemical staining revealed that RORγt-expressing cells infiltrated in the border zone after MI. Flow cytometric analysis showed that RORγt-expressing cells were released from the spleen at day 1 after MI. Though RORγt-expressing cells in spleen expressed γδTCR or CD4, γδTCR^+^ cells were major population of RORγt-expressing cells that infiltrated into post-infarct myocardium. To address the biological functions of RORγt-expressing cells in infarcted hearts, we used mice with *enhanced GFP* gene heterozygously knocked-in at *RORγt* locus (RORγt^+/-^ mice), which physiologically showed reduced expression of RORγt mRNA in thymus. Kaplan-Meier analysis showed that MI-induced mortality was higher in RORγt^+/-^ mice than wild-type (WT) mice. Masson’s trichrome staining demonstrated that cardiac injury was exacerbated in RORγt^+/-^ mice 7 days after MI (Injured area: RORγt^+/-^; 42.1±6.5%, WT; 34.0±3.7%, circumference of injured myocardium: RORγt^+/-^; 61.8±4.8%, WT; 49.6±5.1%), accompanied by exacerbation of cardiac function (fractional shortening: RORγt^+/-^; 32.9±2.9%, WT; 38.3±3.6%). Moreover, immunohistochemical analyses revealed that capillary density in border zone was significantly reduced in RORγt^+/-^ mice after MI, compared with WT mice, associated with the reduced expression of angiopoietin 2. Finally, the mRNA expression of RORγt, IL-17A, IL-17F and IL-23 receptor (IL-23R) mRNA and protein expression of IL-10 were decreased in RORγt^+/-^ hearts.

**Conclusions:**

Heterozygous deletion of *RORγt* gene resulted in aggravated cardiac remodeling, accompanied by reduced capillary density, after MI, suggesting that RORγt-expressing cells contribute to tissue repair in infarcted myocardium.

## Introduction

Myocardial infarction (MI) is one of the major causes of heart failure. MI induces cardiomyocyte death, followed by infiltration of immune cells into post-infarct myocardium. The immune cells positively or negatively regulate myocardial inflammation and modulate adverse cardiac remodeling [1–3]. For instance, neutrophils infiltrate into infarcted myocardium immediately after coronary occlusion and aggravate tissue damage by producing reactive oxygen species (ROS) [4,5]. In contrast, macrophages contribute to the clearance of dead cells after MI and promote angiogenesis, leading to wound healing [6–8]. Therefore, in order to understand the importance of immune cells in cardiac remodeling, it would be required to make clear the biological functions of immune cells based on cell-lineage in more detail.

The retinoic acid receptor-related orphan nuclear receptor γt (RORγt) was originally identified as an essential transcription factor for IL-17A producing T cell differentiation [9,10]. Physiologically, IL-17A plays important roles in host protection against the microbial infection in the bowels [11–13]. IL-17A is also implicated in the pathogenesis of various inflammatory diseases such as atherosclerosis [14,15], psoriasis [16,17] and autoimmune diseases [18,19]. Interestingly, recent studies have proposed that the deficiency of IL-17A could ameliorate left ventricular remodeling after MI [20,21], suggesting that IL-17A is detrimental to the maintenance of cardiac homeostasis after MI; however, it is required to clarify the biological significance of RORγt-expressing cells that produce IL-17A, because IL-17A is a pleiotropic cytokine and locally functions in various tissues.

RORγt-expressing cells express various cytokines and cytokine receptors, including IL-17F and IL-23R, as well as IL-17A [22,23]. In this study, we addressed the pathophysiological roles of RORγt-expressing cells in cardiac remodeling after MI, using mice with *enhanced GFP* (*eGFP*) gene heterozygously knocked-in at *RORγt* locus (RORγt^+/-^ mice), because homozygous deletion of RORγt gene showed higher mortality by coronary ligation, compared with WT or RORγt^+/-^ mice, in our preliminary study. In RORγt^+/-^ mice, the myocardial expression of RORγt and IL-17A was reduced in the heart after MI, compared with wild-type (WT) mice. Importantly, post-infarct cardiac remodeling was exacerbated in RORγt^+/-^ mice, accompanied by reduced capillary density, suggesting that RORγt-expressing cells ameliorate cardiac remodeling after MI. The present study is the first demonstration that RORγt-expressing cells exhibit cardioprotective properties after MI.

## Materials and methods

### Animal care

Animal care was performed according to the Osaka University animal care guidelines. The study was approved by the Institutional Animal Care and Use Committee of the Graduate School of Pharmaceutical Science, Osaka University (Permit Number; DOUYAKU 26–4). All animal experiments were in accordance with the Guide for the Care and Use of Laboratory Animals, Eighth Edition, updated by the US National Research Council Committee in 2011. Male C57BL/6 mice (8–12 weeks old, 25-30g body weight) were purchased from Japan SLC, Inc, and maintained on a 12 h light/dark cycle with free access to food and water at Animal Care Facility of Graduate School of Pharmaceutical Sciences, Osaka University. At the endpoints of all experiments, the hearts were excised from isoflurane-anesthetized mice. All efforts were made to minimize suffering.

### Induction of MI

Murine MI was induced by permanent ligation of the left anterior descending coronary artery as previously described [24].

### Splenectomy surgery

Mice were ventilated and anesthetized by isoflurane. After the left-side small laparotomy, splenic vessels were ligated with 7–0 silk sutures and the spleen was removed. The peritoneum and skin were closed with 5–0 silk sutures. MI operation was performed immediately after the splenic removal.

### RORγt-enhanced green fluorescence protein (eGFP) transgenic mice

The genetically engineered mice with *eGFP* cDNA knocked-in at *ROR*γ*t* gene locus on a C57BL/6 background, designated as RORγt^+/-^ mice, were purchased from Jackson Laboratories (stock number: 007572). Genomic DNA was prepared from RORγt WT, RORγt^+/-^, and RORγt^-/-^ mice. The genotyping of RORγt and eGFP was performed by PCR using specific primers. The primers used for genotyping were as follows: RORγt and eGFP forward 5’- GCC ACC TGT GTG GAG CAG AGC TTA -3’, RORγt reverse 5’- GGA TGC CCC CAT TCA CTT ACT TCT -3’, eGFP reverse 5’- TCC TTG AAG AAG ATG GTG CG -3’. Because, in our preliminary study, RORγt^-/-^ mice showed higher mortality compared with WT or RORγt^+/-^ mice within 2 days after MI (WT: 7.1%, RORγt^+/-^: 5.3%, RORγt^-/-^: 25.0%). RORγt^+/-^ mice were used to evaluate the pathophysiological roles of RORγt-expressing cells in cardiac remodeling.

### Quantitative RT-PCR

Total RNA sample was prepared using QIAzol reagent (QIAGEN). RT-PCR was performed according to the manufacturer’s protocol, as described previously [25]. The expression of RORγt, VEGF, CXCL5, angiopoietin 2, IL-17A, IL-17F, IL-23R, IL-6, IL-1β, TNF-α, IFN-γ, IL-10, and TGF-β mRNA in the left ventricle (LV) from the ligation point to the apex was estimated by real-time PCR using the SYBR green system (Applied Biosystems). The expression of GAPDH was measured and used as an internal control. The primers used in this study are shown in Supplementary material online, S1 Table.

### Histological analysis

Cardiac injury was histologically estimated as described previously [24,26]. Briefly, hearts were harvested at day 7 after MI. The hearts were sliced from the position about 300 μm distal to the ligation point at 5-μm thickness, followed by staining with Masson’s trichrome method. The area or circumference ratio of the injured region to LV was measured using Image J software (National Institutes of Health) by a researcher who was blinded to the experimental condition.

Immunohistochemical analysis was performed as described previously [24]. In brief, frozen sections were fixed with 4% paraformaldehyde (PFA) or acetone. RORγt-expressing cells infiltration was stained using Histofine Mousestain kit (Nichirei Bioscience) with mouse anti-RORγt monoclonal antibody (Clone: Q31-378, BD Bioscience). Mouse non-immune IgG (Santa Cruz Biotechnology) was used as control. Nuclei were counterstained with hematoxylin. To estimate the number of RORγt-expressing cells, photomicrographs of border area and remote area were taken at random. Border area was defined as the region < 1 mm from the end of infarct area. RORγt-positive cells were counted in number by the researcher who was blinded to the assay condition.

To estimate the capillary density, capillary vessels were stained using a Vectastain ABC kit (Vector Laboratories) with rat anti-CD31 monoclonal antibody (Clone: MEC13.3, BD Bioscience). Sections were photographed at border area, and then CD31-positive cells were counted in number by the researcher who was blinded to the assay condition.

### Flow cytometric analysis

Spleens were harvested, triturated, and passed through a 70 μm cell strainer in PBS containing 3% fetal bovine serum (FBS). Cells were treated Lysing buffer (BD Bioscience) to exclude erythrocytes. To block Fc receptors, cells were incubated with rat anti-CD16/32 monoclonal antibody (Clone: 93, BioLegend) on ice for 15 minutes. Cells were stained surface markers with PE-conjugated armenian hamster anti-γδTCR monoclonal antibody (Clone: GL3, BioLegend) and FITC-conjugated rat anti-CD4 monoclonal antibody (Clone: RM4-5, BD Bioscience) on ice for 40 minutes. Transcription Factor Staining Buffer Set (eBioscience) was used for intracellular staining according to the manufacturer's protocol. After fixation and permeabilization, cells were stained with APC-conjugated rat anti-RORγt antibody (Clone: B2D, eBioscience) on ice for 40 minutes. Isotype-control staining was performed using FITC- or APC-conjugated rat control IgG (Clone: RTK2758, BioLeged) and PE-conjugated rat control IgG (Clone: R35-95, BD Bioscience). Flow cytometric analysis was performed on FACS Aria II (BD Bioscience) and analyzed using BD FACS Diva software (BD Bioscience).

### Analysis of cardiac function by echocardiography

Echocardiography was performed as described previously [25].

### Gravimetric analysis

Mice were weighted at day 7 after MI. The heart and lung were harvested after euthanasia by inhalation of isoflurane and washed with cold PBS. The weight of these organs was measured and normalized with body weight.

### Enzyme-linked immunosorbent assay

Heart homogenates were prepared and IL-10 concentration in myocardium was evaluated with ELISA kit (BioLegend, ELISA MAX^TM^ Deluxe Set#431414).

### Statistical analysis

Data are shown as means ± SD. Survival rate was analyzed by the Kaplan-Meier method using a log-rank test. Comparisons between two groups were performed with the use of Student *t*-test. One-way ANOVA followed by a Tukey-Kramer or Dunnett test was used for multiple comparisons. *P* value < 0.05 was considered to be statistically significant.

## Results

### RORγt-expressing cells infiltrated in the heart after MI

**Fig 1:**
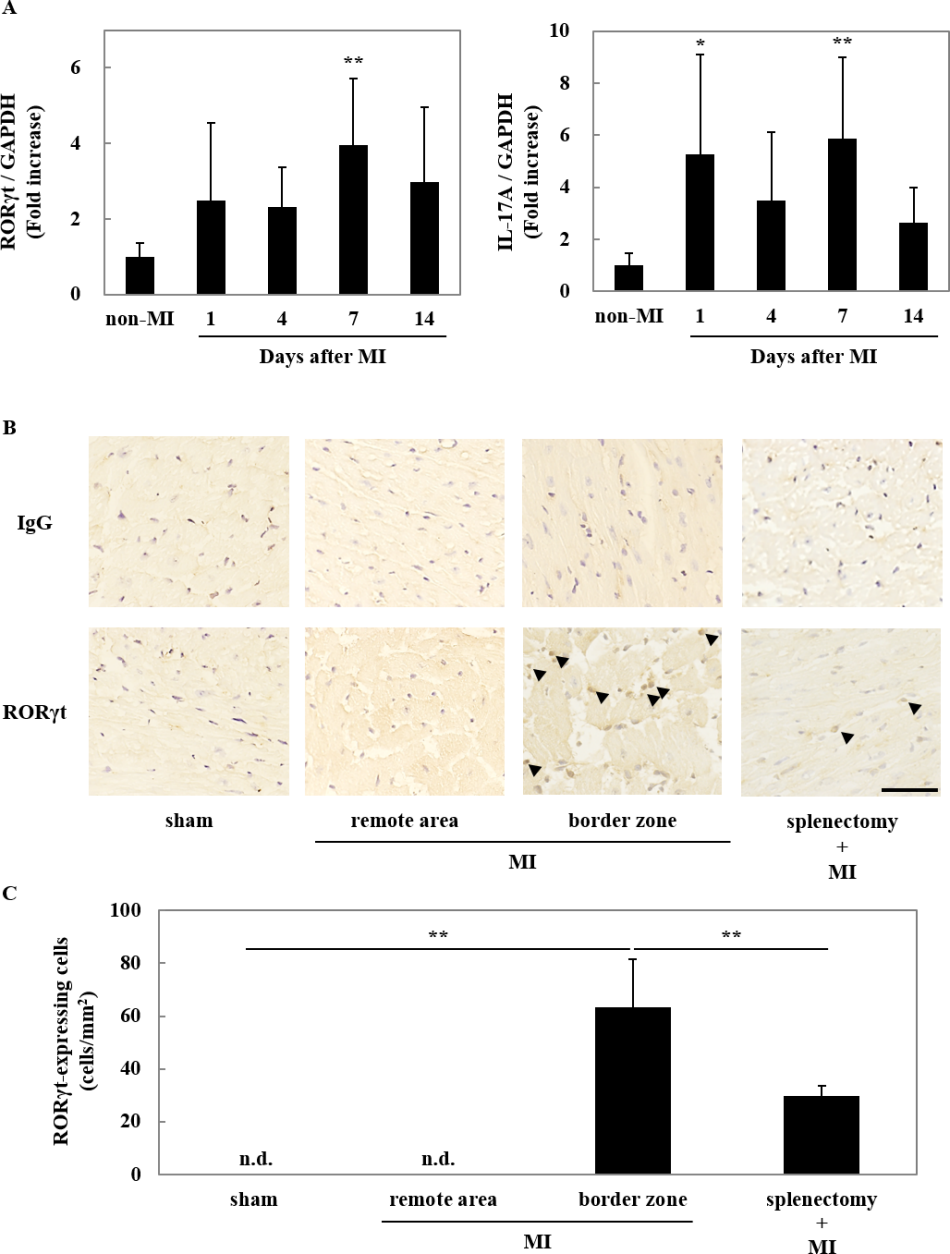
RORγt-expressing cells infiltrated in the heart after MI. (A) C57BL/6 mice were subjected to MI operation, and hearts were harvested at the indicated time points. Total RNA was prepared from the ligation point to the apex of hearts, and quantitative RT-PCR was performed for RORγt (left) and IL-17A (right). The expression of RORγt and IL-17A mRNA was normalized with that of GAPDH and were shown as a ratio to the average value of non-MI. Data are shown as means ± SD; n = 7 for each condition. *P < 0.05 and **P < 0.01 vs non-MI by one-way ANOVA followed by Dunnett test. (B) The sections were prepared from the hearts at day 7 after MI, MI plus splenectomy or sham operation. Immunohistochemical analyses were performed with anti-RORγt antibody or mouse non-immune IgG as a control. Nuclei were stained by hematoxylin (Blue). Representative images at border zone and remote area are shown. The arrowhead indicates RORγt-positive cell (Brown). Bar = 50 μm. (C) Quantitative analyses of RORγt-expressing cells. Thirty visual fields at the border zone or remote area were photographed from 5 hearts. Cells that were positively stained with anti-RORγt at nuclei were counted. Data are shown as means γ SD. n.d., not detectable, **P < 0.01 by one-way ANOVA followed by Turkey-Kramer test.

We examined the expression of RORγt and IL-17A mRNA in post-infarct myocardium by quantitative RT-PCR (Fig 1A). After C57BL/6 mice were subjected to MI, mRNA expression was measured at various time points. The expression of RORγt and IL-17A transcripts was upregulated at day 1 and the expression of these genes displayed its peak at day 7 after MI. Consistently, immunohistochemical staining with anti-RORγt antibody revealed that RORγt-expressing cells infiltrated in the border area after MI, but not in remote area or sham operated hearts (Fig 1B and 1C). Previously, we demonstrated that RORγt-expressing cells are required for the onset of experimental autoimmune myocarditis (EAM) [27]. Interestingly, it is reported that EAM is produced by adoptive transfer of splenocytes after MI [28]. Therefore, we examined the effects of the splenectomy on the infiltration of RORγt-expressing cells into post-infarct myocardium and found that the number of RORγt-expressing cells infiltration was reduced by splenectomy surgery (Fig 1B and 1C). These data indicate that RORγt-expressing cells were released at least partially from the spleen. It should be noted that the infiltration of RORγt-expressing cells was not completely inhibited by splenectomy, suggesting that some fraction of RORγt-expressing cells were originated from other lymphoid tissues, such as lymph nodes.

### RORγt-expressing cells were released from the spleen after MI

**Fig 2:**
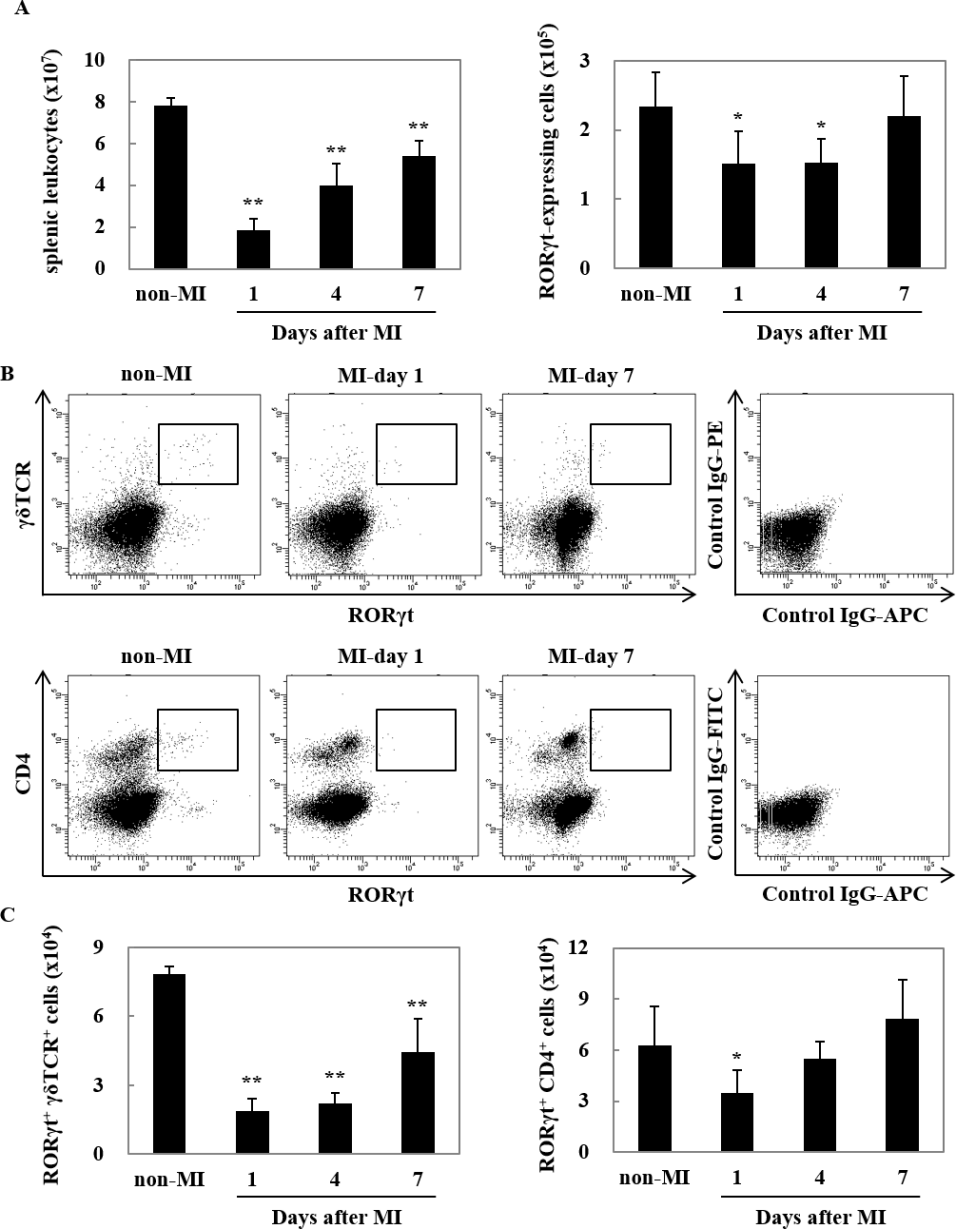
RORγt-expressing cells were released from spleen after MI. (A) Quantitative analyses of the number of splenic leukocytes (left) and RORγt-expressing cells (right). Data are shown as means ± SD; n = 5 for non-MI, 6 for each MI conditions. *P < 0.05, **P < 0.01 by one-way ANOVA followed by Dunnett test. (B) γδTCR^+^ RORγt^+^ cells (upper) and CD4^+^ RORγt^+^ cells (bottom) were detected by flow cytometric analysis. Blots are representative of 4 independents experimental conditions. (C) Quantitative analyses of the number of γδTCR^+^ RORγt^+^ cells (left) and CD4^+^ RORγt^+^ cells (right). Data are shown as means ± SD; n = 5 for non-MI, 6 for each MI groups. *P < 0.05, **P < 0.01 vs non-MI by one-way ANOVA followed by Dunnett test.

Since splenectomy reduced the number of RORγt-expressing cells that infiltrated post-infarct myocardium, we performed flow cytometric analysis for splenic leukocytes to characterize RORγt-expressing cells. Consistent with RORγt-expressing cells infiltration in infarcted heart, the number of leukocytes and RORγt-expressing cells in the spleen was reduced at day 1 after MI (Fig 2A). The staining of cell surface markers revealed that RORγt-expressing cells consist of RORγt^+^ γδTCR^+^ cells and RORγt^+^ CD4^+^ cells (Fig 2B). We analyzed temporal changes of both RORγt^+^ γδTCR^+^ cells and RORγt^+^ CD4^+^ cells in the spleen after MI. RORγt^+^ γδTCR^+^ cells were more abundantly released from the spleen from day 1 to day 7 after MI, while RORγt^+^ CD4^+^ cells were transiently released at day1 (Fig 2C). Considering that the expression of RORγt increased in the heart from day 1 to day 7 after MI, it is likely that majority of RORγt-expressing cells in post-infarct myocardium are RORγt^+^ γδTCR^+^ cells.

### Heterozygous deletion of RORγt gene increased the mortality after MI

**Fig 3:**
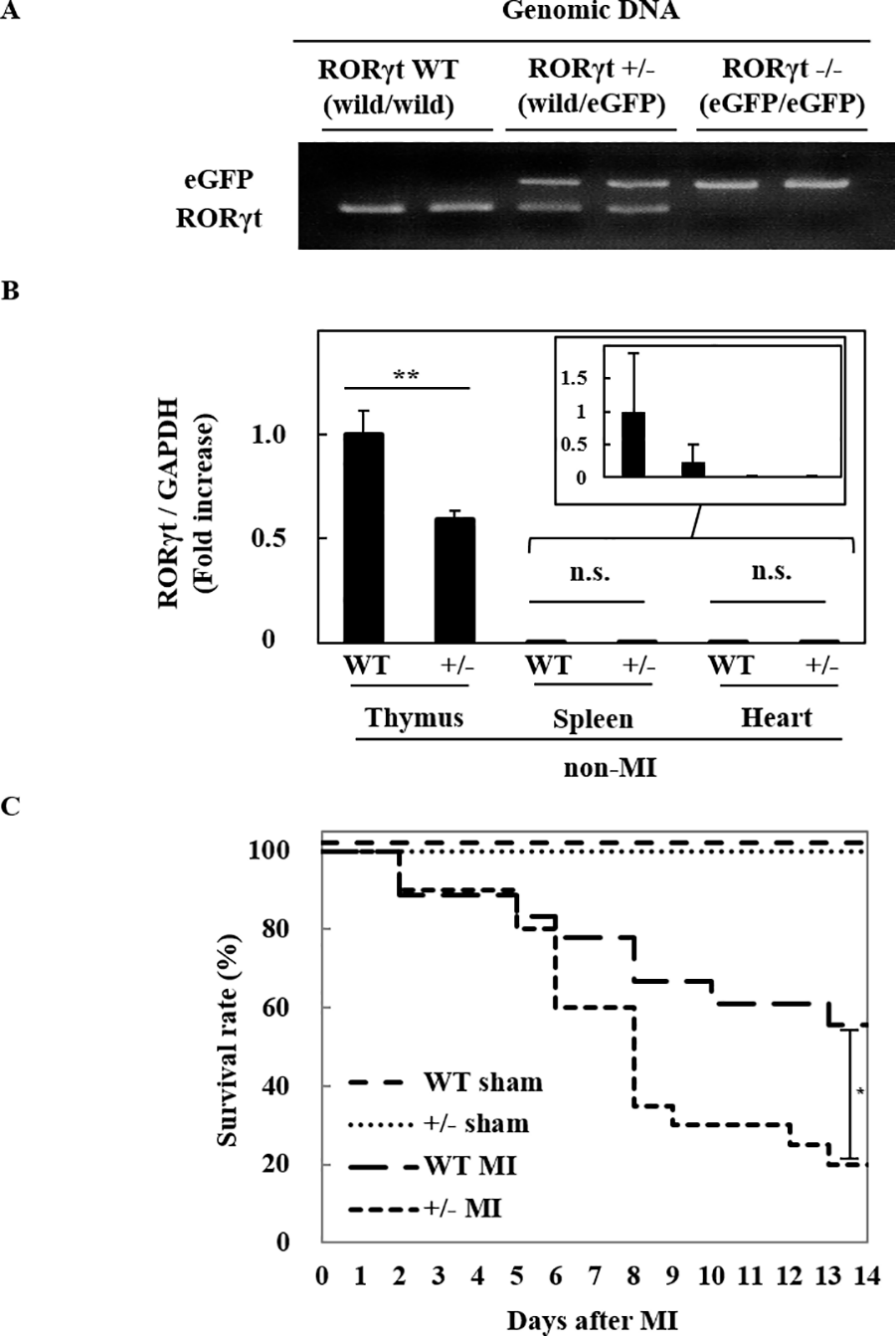
Heterozygous deletion of RORγt gene increased the mortality after MI. (A) The representative image of PCR products of RORγt and eGFP from genomic DNA. (B) Total RNA was prepared from thymus, spleen and heart of RORγt^+/-^ or WT mice. Quantitative RT-PCR was performed for RORγt. The expression of RORγt mRNA was normalized with that of GAPDH. The expression levels were calculated as a ratio to the average value of thymus on WT mice. Data are shown as means ± SD; n = 3 for thymus of WT and RORγt^+/-^ mice, heart of WT and RORγt^+/-^ mice, spleen of RORγt^+/-^ mice, n = 6 for spleen of WT mice. **P < 0.01 vs WT mice by Student’s *t*-test. (C) Kaplan-Meier analyses of survival rate after MI or sham operation. Mice were subjected to MI or sham operation. n = 18 for WT mice with MI, 20 for RORγt^+/-^ mice with MI, 6 for each group with sham operation. The survival rate after MI or sham operation was estimated by the Kaplan-Meier method. *P < 0.05 vs WT mice by log-rank test.

To analyze the pathophysiological significance of RORγt-expressing cells in post-infarct myocardium, we used RORγt^+/-^ mice with *eGFP* gene heterozygously knocked-in at *RORγt* gene locus (Fig 3A). In order to confirm that *RORγt* gene was successfully reduced in RORγt^+/-^ mice, we examined physiological expression of RORγt mRNA in the thymus by quantitative RT-PCR analysis, because RORγt mRNA is constitutively expressed in the thymus [9]. The RORγt mRNA expression was significantly suppressed in the thymus in RORγt^+/-^ mice compared with WT mice (Fig 3B). Similarly, the expression of RORγt in spleen has tendency to decrease in RORγt^+/-^ mice with wide standard deviation, possibly because splenic cells are composed of more various immune cells than thymic cells. In comparison with the thymus, the expression of RORγt mRNA in non-MI hearts was very low in both RORγt^+/-^ and WT mice. Consistently, echocardiographic analysis showed that there was no difference in cardiac function between these two groups of mice before MI (Table 1). However, after MI, RORγt^+/-^ mice showed higher rate of mortality (Fig 3C), accompanied by aggravated cardiac dysfunction at day 7 after MI, compared with WT mice (Table 1).

**Table 1:** Echocardiographic analyses on the cardiac function.

Days after MI	0		2		7	
	WT	RORγt^+/-^	WT	RORγt^+/-^	WT	RORγt^+/-^
FS (%)	51.6±3.2	52.0±2.7	46.5±3.1^§^	44.8±1.6^‡^	38.3±3.6^§^	32.9±2.9^‡^*
HR (bpm)	520.4±56.1	539.4±46.2	486.2±96.2	471.7±50.1	529.8±57.9	487.8±55.8
LVIDd (mm)	3.7±0.3	3.4±0.3	4.2±0.4	4.0±0.4	4.9±0.7^§^	4.9±0.5^‡^
LVIDs (mm)	1.8±0.2	1.8±0.2	2.2±0.3	2.2±0.3	3.0±0.5^§^	3.3±0.4^‡^

FS; Fractional Shortening, HR; Heart Rate, LVIDd; Left Ventricular Internal Dimension in diastole, LVIDs; Left Ventricular Internal Dimension in systole. Data are shown as mean ± S.D; n = 14 for day 0-WT mice, 11 for day 0-RORγt^+/-^ mice, 17 for day 2-WT mice, 11 for day 2-RORγt^+/-^ mice, 17 for day 7-WT mice, 13 for day 7-RORγt^+/-^ mice.

*P<0.01 vs day 7-WT mice

^**§**^*P*<0.01 vs day 0-WT mice

^**‡**^*P*<0.01 vs day 0-RORγt^+/-^ mice by one-way ANOVA followed by Tukey-Kramer test.

### Adverse cardiac remodeling was exacerbated in RORγt^+/-^ mice after MI

**Fig 4:**
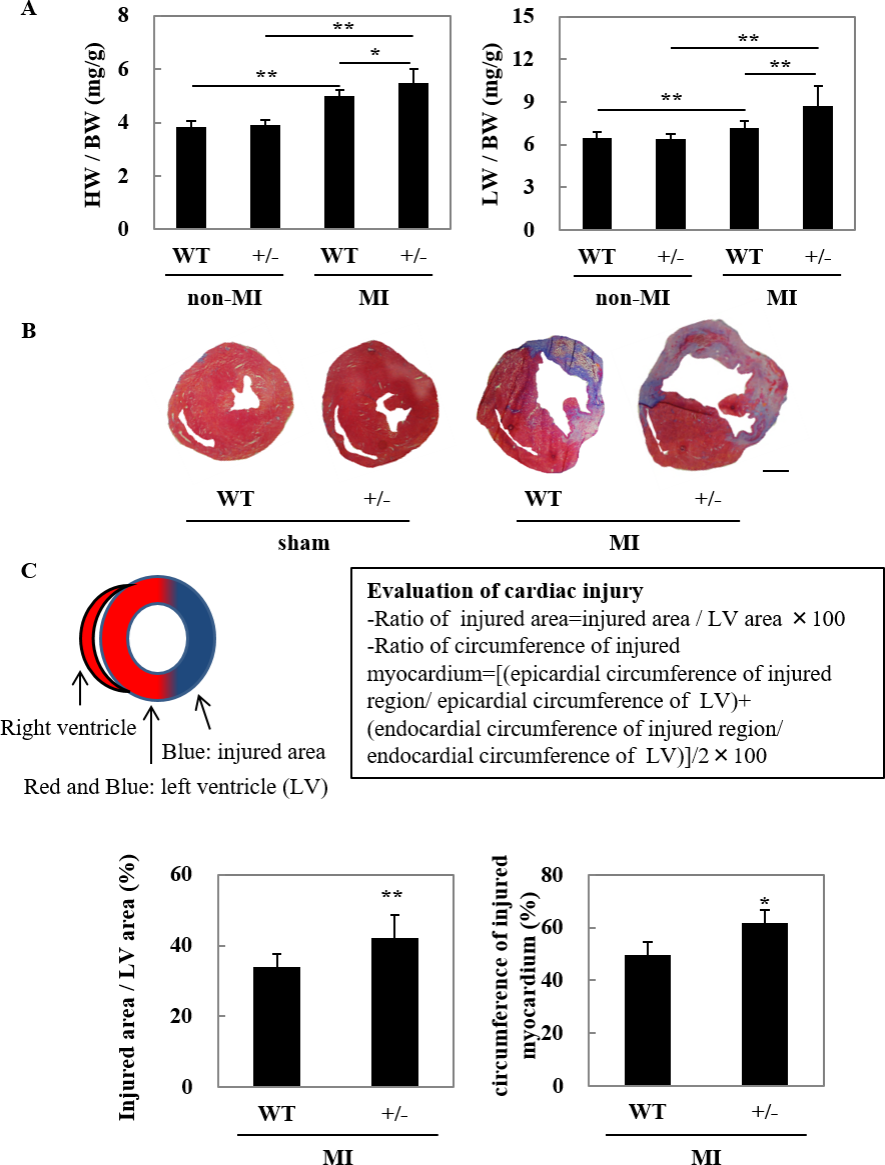
Adverse cardiac remodeling was exacerbated in RORγt^+/-^ mice after MI. (A) The heart weight-to-body weight ratio (HW/BW, left) and the lung weight-to-body weight ratio (LW/BW, right) were estimated. Data are shown as means ± SD; n = 8 for WT mice with non-MI, 7 for RORγt^+/-^ mice with non-MI, 8 for WT mice with MI, 18 for RORγt^+/-^ mice with MI. *P < 0.05 and **P < 0.01 by Turkey-Kramer test. Heart sections were prepared at day 7 after MI and stained with Masson’s trichrome method. (B) Representative images. Bar = 1 mm. (C) Upper, the image and method for analyzing the cardiac injury. Lower-left, the ratio of injured area to left ventricular (LV) area of MI operated mice. Lower-right, the ratio of injured circumference to LV circumference of MI operated mice. Data are shown as means ± SD; n = 15 for WT mice, 11 for RORγt^+/-^ mice. **P < 0.01 vs WT mice by Student’s *t*-test.

To make clear the effects of RORγt knockdown on adverse cardiac remodeling after MI, we conducted gravimetric analysis and measured severity of cardiac injury in RORγt^+/-^ and WT mice (Fig 4). In this study, we analyzed cardiac remodeling at day 7 after MI, because approximately 80% of RORγt^+/-^ mice died by day 14 (Fig 3C). Consistent with the aggravated cardiac dysfunction in RORγt^+/-^ mice at day 7 after MI, the ratio of heart weight to body weight and lung weight to body weight were both increased in RORγt^+/-^ mice after MI, compared with WT mice (Fig 4A). Histological analyses by Masson’s trichrome staining revealed that severity of cardiac injury, including cardiac fibrosis, was exacerbated in RORγt^+/-^ mice after MI, although there was no histological difference between RORγt^+/-^ mice and WT mice in sham-operated groups (Fig 4B). We quantified injured area and circumference, and found that cardiac injury was significantly increased in RORγt^+/-^ mice (Fig 4B and 4C).

### Capillary density was reduced in RORγt^+/-^ hearts after MI

**Fig 5:**
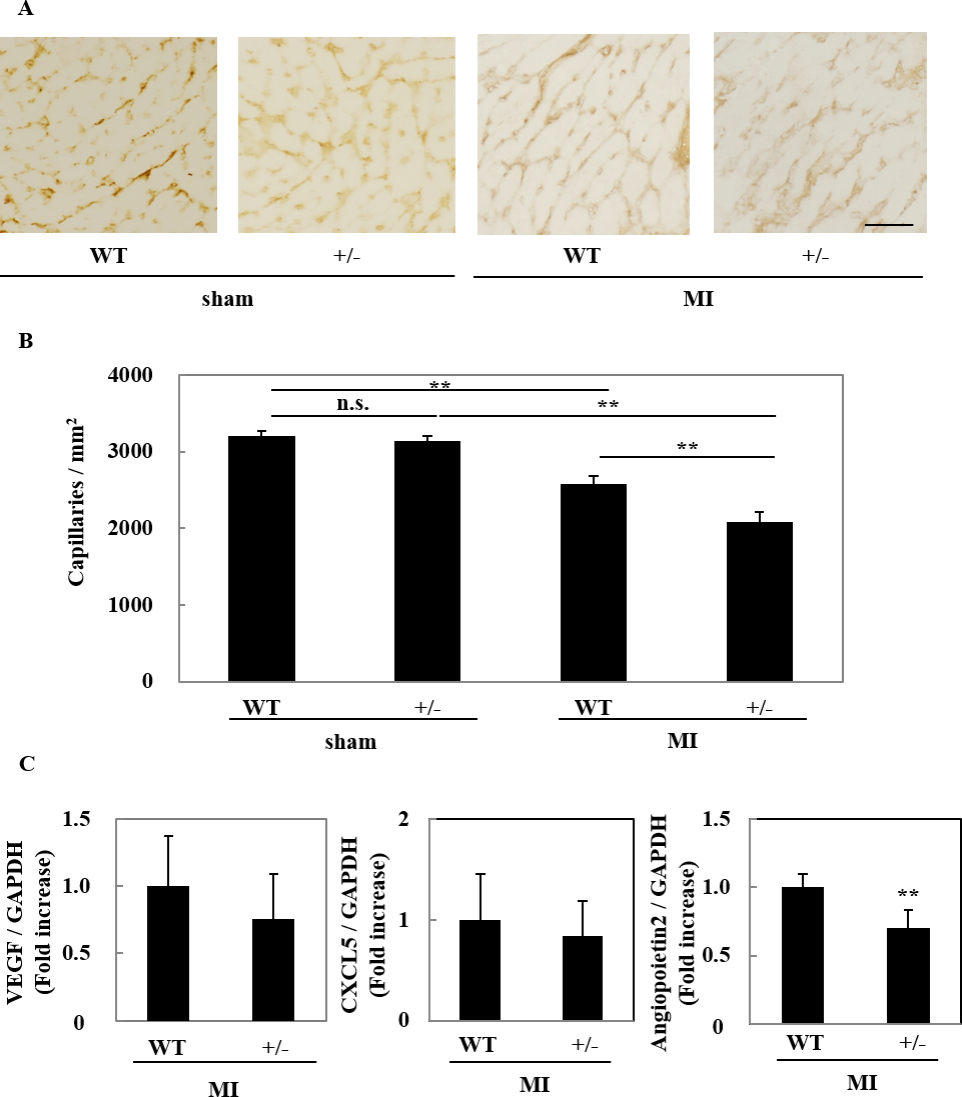
Capillary density was reduced in RORγt^+/-^ hearts after MI. (A) Capillaries in the heart were detected by immunohistochemical staining with anti-CD31 antibody. Representative images at border zone are shown. Bar = 50 µm. (B) Sixty visual fields at the border zone from 5 hearts were randomly selected. The number of CD31-positive cells was counted to calculate the capillary density. Data are shown as means ± SD. **P < 0.01 by one-way ANOVA followed by Turkey-Kramer test. (C) Quantitative RT-PCR was performed for VEGF, CXCL5 and angiopoietin 2 at day 4 after MI. The expression of the transcripts was normalized with that of GAPDH and shown as the ratio to the average value of WT mice. Data are shown as means ± SD; n = 4. **P < 0.01 vs WT mice by Student’s *t*-test.

Impaired vascular formation in post-infarct myocardium aggravates adverse cardiac remodeling [29,30]. Therefore, we measured capillary density at day 7 after MI by immunohistochemical staining with anti-CD31 antibody (Fig 5). In the border zone, capillary density was significantly reduced in RORγt^+/-^ mice relative to WT mice, whereas there was no difference between WT and RORγt^+/-^ mice in sham-operation. To explore the mechanisms of impaired angiogenesis in RORγt^+/-^ mice, the mRNA expression of angiogenetic factors, such as VEGF, CXCL5 and angiopoietin 2, was measured by quantitative PCR. While VEGF and CXCL5 mRNA expressions were unchanged between RORγt^+/-^ mice and WT mice, angiopoietin 2 expression was significantly reduced in RORγt^+/-^ mice at day 4 after MI (Fig 5C).

### Effects of heterozygous RORγt deletion on cytokine production in post-infarct myocardium

**Fig 6:**
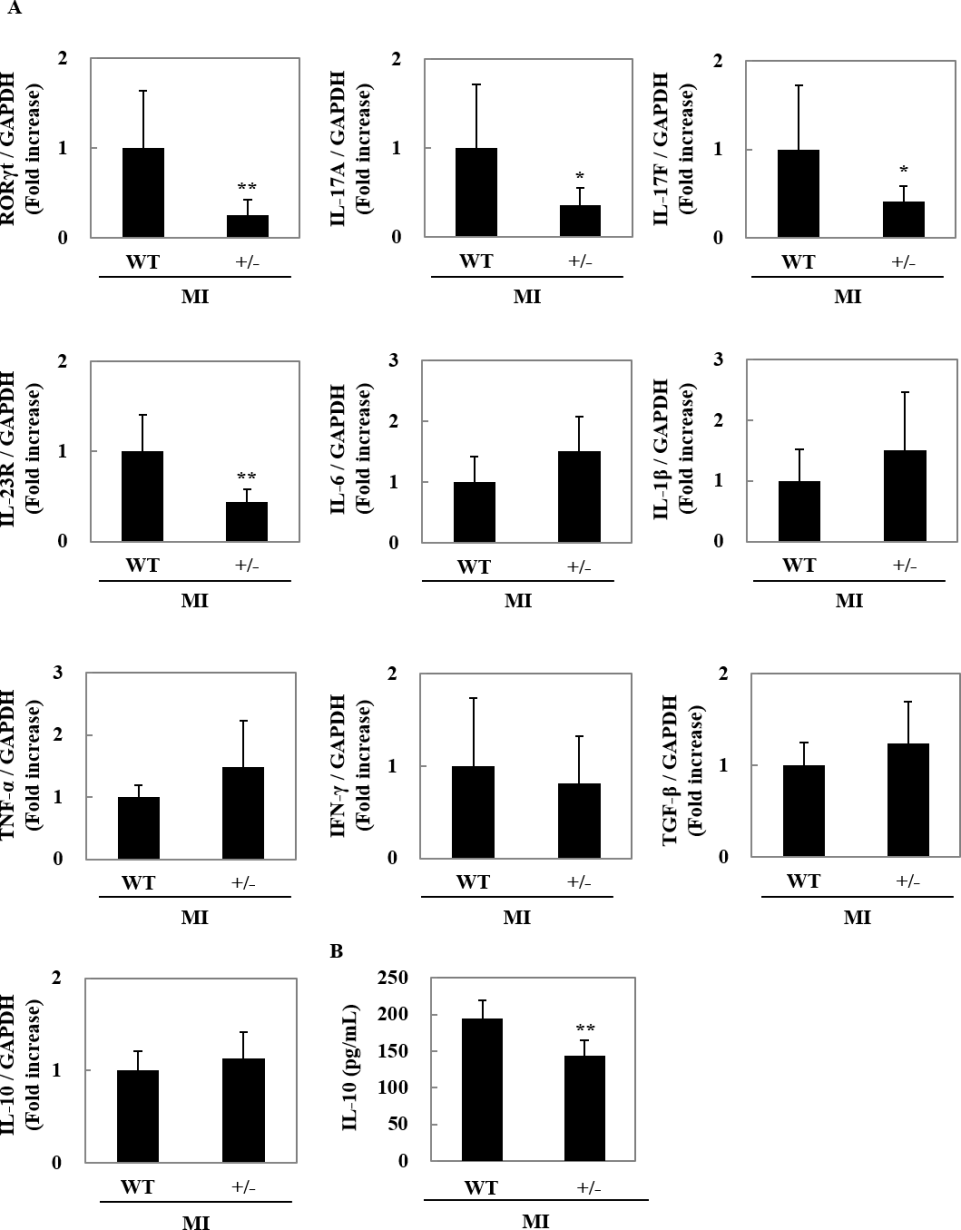
Effects of heterozygous RORγt deletion on cytokine production in post-infarct myocardium. (A) RORγt^+/-^ mice and WT mice were subjected to MI operation, and hearts were harvested at day 7 after MI. Total RNA was prepared from hearts from the ligation point to the apex, and quantitative RT-PCR was performed for RORγt, IL-17A, IL-17F, IL-23R IL-6, IL-1β, TNF-α, IFN-γ, IL-10 and TGF-β mRNA levels. The expressions of the transcripts were normalized with that of GAPDH and shown as the ratio to the average value of WT mice. Data are shown as means ± SD; n = 13 for WT mice, 8 for RORγt^**+/-**^ mice. *P < 0.05 and **P < 0.01 vs WT mice by Student’s *t*-test. (B) Heart homogenate was prepared from infarct hearts of RORγt^+/-^ mice and WT mice at day 7 after MI. ELISA was performed to evaluate the concentration of IL-10 protein. Data are shown as means ± SD; n = 6 for WT mice, n = 7 for RORγt+/- mice. **P < 0.01 vs WT mice by Student’s *t*-test.

The inflammatory reaction is closely associated with the progression of cardiac remodeling. Therefore, to assess the effects of heterozygous RORγt deletion on cytokine production, we performed quantitative RT-PCR and estimated mRNA expression of cytokines and their related genes (Fig 6). The myocardial expression of RORγt, IL-17A, IL-17F, and IL-23R mRNA was significantly suppressed in RORγt^+/-^ mice at day 7 after MI, compared with WT mice, respectively. Consistent with previous reports that IL-17 was thought to be only increased locally in the infarcted region after MI [31,32], there was no significant difference in serum IL-17 level between RORγt^+/-^ and WT mice after MI (S1 Fig). Moreover, the expression of pro-inflammatory cytokines, including IL-6, IL-1β, TNF-α, and IFN-γ, as well as anti-inflammatory cytokines, including IL-10 and TGF-β, was not influenced by heterozygous deletion of *RORγt* gene. Whereas, ELISA revealed that IL-10 protein in myocardium was reduced in RORγt^+/-^ mice in comparison to control mice at day7 after MI.

## Discussion

In post-infarct myocardium, immune cells positively or negatively modulate cardiac remodeling [1–8]. Here, we have demonstrated that RORγt-expressing cells infiltrated into myocardium after MI. To analyze the pathophysiological roles of RORγt-expressing cells, MI was generated in RORγt^+/-^ mice with *eGFP* gene heterozygously knocked-in at RORγt locus. In RORγt^+/-^ mice, the mortality was increased with reduced expression of RORγt and IL-17A transcripts in post-infarct myocardium. Importantly, adverse cardiac remodeling was exacerbated in RORγt^+/-^ mice, accompanied by reduced capillary density. These data suggest that RORγt-expressing cells play cardioprotective roles in cardiac remodeling after MI.

In this study, we used RORγt^+/-^ mice instead of RORγt-deficient mice, because RORγt^-/-^ mice showed higher mortality immediately after MI. RORγt^+/-^ mice grew normally and echocardiography showed that there was no difference in cardiac function between RORγt^+/-^ and WT mice under physiological conditions. We confirmed that the expression level of RORγt transcript in the thymus was reduced by 41% in RORγt^+/-^ mice, compared with WT, indicating that RORγt^+/-^ mice could be used as RORγt knock-down model.

RORγt was originally identified as a transcription factor responsible for differentiation of IL-17-producing T cells [9,10]. Previously, we have demonstrated that RORγt is essential for the induction of experimental autoimmune myocarditis (EAM) [27]; however, the pathophysiological roles of RORγt-expressing cells poorly understand in cardiac remodeling after MI. RORγt is a transcriptional factor for various cytokines and their receptors, including IL-17A, IL-17F and IL-23R [22,23]. Indeed, the expression of these cytokines and cytokine receptors was reduced in post-infarct myocardium in RORγt^+/-^ mice. Interestingly, we showed that heterozygous deletion of *RORγt* gene exacerbated adverse cardiac remodeling after MI, suggesting that RORγt-expressing cells could prevent cardiac remodeling.

Though RORγt is essential for Th17 differentiation, RORγt expression is not limited to Th17 cells [22,23]. Our data presented here propose that RORγt-mediated regulation of inflammatory reaction could be crucial for protection from adverse cardiac remodeling, independently of Th17 cells. Importantly, we demonstrated that RORγt-expressing cells, released from the spleen at day1 to 7 after MI, were γδT cells. Recently, two types of γδT cells have been identified [33]; one is RORγt^+^ IL-17^+^ γδT cell and the other is RORγt^-^ IFN-γ^+^ γδT cell. Consistent with our results, IL-17-producing γδT cells are known to contribute to tissue repair by promoting angiogenesis [34,35].

Recently, it was reported that γδTCR-KO mice exhibited improved survival and cardiac function after MI [20], while we have demonstrated that RORγt-expressing γδTCR^+^ cells play protective roles. The inconsistency between these two studies might be explained by the heterogeneity of γδTCR^+^ cells. As described above, γδTCR^+^ cells consist of at least 2 populations, INF-γ producing γδTCR^+^ cells and IL-17 producing γδTCR^+^ cells. The expression of IL-17 was reduced in RORγt^+/-^ myocardium after MI, while that of INF-γ was not. Therefore, RORγt-expressing γδTCR^+^ cells, observed in this study, are mainly IL-17 producing γδTCR^+^ cells. In other words, the inconsistent results of these two studies might imply that additional subpopulation of γδTCR^+^ cells could exert detrimental effects on cardiac remodeling after MI, though further studies would be needed to identify the subpopulation.

It is interesting that capillary density was reduced in myocardium of RORγt^+/-^ mice after MI. Since angiogenesis prevents post-infarct cardiac fibrosis [29,30], it is proposed that RORγt-expressing cells ameliorated cardiac remodeling at least partially by promoting angiogenesis. This proposal is supported by the finding that the expression of IL-17A and angiopoietin 2 was reduced in post-infarct myocardium in RORγt^+/-^ mice. It is well known that IL-17A promotes vascular formation [36,37]. Moreover, angiopoietin 2 positively regulates angiogenesis in autoimmune diseases [38]. Of course, we cannot exclude the possibility that other secretory factors or cytokines, produced from RORγt-expressing cells, were involved in cardioprotection, because it was reported that deficiency of IL-17A prevented cardiac remodeling and that IL-17A does not necessarily show the beneficial effects on cardiac remodeling [20,21].

In the present study, we have demonstrated that heterozygous ablation of *RORγt* gene exacerbated cardiac remodeling after MI. RORγt-expressing γδT cells exhibited preventive effects against cardiac remodeling at least partially through enhanced revascularization, proposing the cardioprotective property as a novel function of RORγt-expressing γδT cells. Modulation of RORγt^+^ cell-mediated regulation of cardiac remodeling could be a promising therapeutic strategy for cardiovascular diseases.

Limitations: It should be noted that male mice were exclusively used in this study as a limitation. In our previous study [27], we examined the pathophysiological roles of RORγt in experimental autoimmune myocarditis (EAM) and found that RORγt is essential for the onset of EAM. In EAM experiments, we exclusively used male Balb/c mice because EAM can be induced more severely in male mice than in female, as reported previously [39]. Therefore, considering the possibility that the biological function of RORγt is dependent on sex difference and can be more remarkably observed in male mice, we designed this study as an exclusive study of male mice to make clear the biological roles of RORγt in cardiac remodeling after MI.

## Supporting information

S1 Table Primers used in this study. (DOCX)

S1 Fig Serum IL-17 concentration was similar level between RORγt+/- mice and WT mice. (TIF)
