# Supplementary material for: RORγt-expressing cells attenuate cardiac remodeling after myocardial infarction
Source: PLoS One. 2017 Aug 21;12(8):e0183584. doi: 10.1371/journal.pone.0183584 (PMC5565178; doi:10.1371/journal.pone.0183584)
Supplement: S1 Table — (DOCX) [file pone.0183584.s001.docx]

**RORγt-expressing cells attenuate cardiac remodeling after myocardial infarction**

Daichi Enomoto, Kotaro Matsumoto, Tomomi Yamashita, Arisa Kobayashi, Makiko Maeda, Hiroyuki Nakayama, Masanori Obana, Yasushi Fujio

**Supplementary methods**

***ELISA***

At day7 after MI, serum was collected from RORγt^+/-^ mice and WT mice. ELISA assay was performed for IL-17 using BioLegend, ELISA MAX^TM^ Deluxe Set IL-17 (#432505).

**Supplementary figure legends**

**S1 Fig. Serum IL-17 concentration was similar level between RORγt+/- mice and WT mice.**

Serum was collected from RORγt^+/-^ mice and WT mice at day 7 after MI. ELISA assay was performed for IL-17. Data are shown as means ± SD. n = 6.

S1 Table. Primers used in this study

| Genes | Forward Primer |
| --- | --- |
|  | Reverse Primer |
| RORγt | 5’-CCG CTG AGA GGG CTT CAC-3’ |
|  | 5’-TGC AGG AGT AGG CCA CAT TAC A-3’ |
| VEGF | 5’-AGA TCA TGC GGA TCA AAC CTC-3’ |
|  | 5’-GTT CTG TCT TTC TTT GGT CTG C-3’ |
| CXCL5 | 5’-GTT CCA TCT CGC CAT TCA TGC-3’ |
|  | 5’-GCG GCT ATG ACT GAG GAA GG-3’ |
| angiopoietin 2 | 5’-CCT CGA CTA CGA CGA CTC AGT-3’ |
|  | 5’-TCT GCA CCA CAT TCT GTT GGA-3’ |
| IL-17A | 5’-TCA CAC GAG GCA CAA GTG CAC CCA-3’ |
|  | 5’-GTG ACG TGG AAC GGT TGA GGT AGT-3’ |
| IL-17F | 5’-CCC ATG GGA TTA CAA CAT CAC TC-3’ |
|  | 5’-CAC TGG GCC TCA GCG ATC-3’ |
| IL-23R | 5’-TCA GTG CTA CAA TCT TCA GAG GAC A-3’ |
|  | 5’- GCC AAG AAG ACC ATT CCC GA-3’ |
| IL-6 | 5’-AAG AGA CTT CCA TCC AGT TGC CTT C-3’ |
|  | 5’-ATT ATA TCC AGT TTG GTA GCA TCC ATC-3’ |
| IL-1β | 5’-GCA GCT ATG GCA ACT GTT CCT-3’ |
|  | 5’-ATG AGT GTA ACT GCC TGC CTG-3’ |
| TNF-α | 5’-CAA AACT TCG AGT GAC AAG CCT GTA GC-3’ |
|  | 5’-CCC TTG AAG AGA ACC TGG GAG TAG A-3’ |
| IFN-γ | 5’-GCC ATC AGC AAC AAC ATA AGC G-3’ |
|  | 5’-GGG TTG TTG ACC TCA AAC TTG G-3’ |
| IL-10 | 5’-AGC CGG GAA GAC AAT AAC TG-3’ |
|  | 5’-TCC AGC TGG TCC TTT GTT TG-3’ |
| TGF-β | 5’-CCT GAG TGG CTG TCT TTT GAC G-3’ |
|  | 5’-AGT GAG CGC TGA ATC GAA AGC-3’ |
| GAPDH | 5’-CAT CAC CAT CTT CCA GGA GCG-3’ |
|  | 5’-GAG GGG CCA TCC ACA GTC TTC-3’ |
