# Supplementary figures and images for: RORγt-expressing cells attenuate cardiac remodeling after myocardial infarction
Source: PLoS One. 2017 Aug 21;12(8):e0183584. doi: 10.1371/journal.pone.0183584 (PMC5565178; doi:10.1371/journal.pone.0183584)

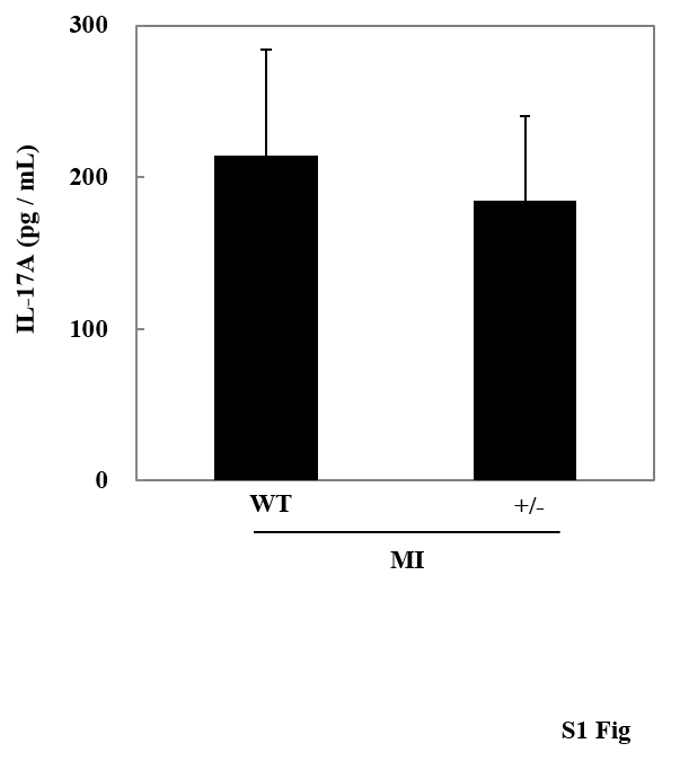

Supplement: S1 Fig — (TIF) [file pone.0183584.s002.tif]
